# Supplementary material for: StudyMe: a new mobile app for user-centric N-of-1 trials
Source: Trials. 2022 Dec 26;23:1045. doi: 10.1186/s13063-022-06893-7 (PMC9793632; doi:10.1186/s13063-022-06893-7)
Supplement: Supplementary file 1 — Additional file 1: Supplementary Text S1. Details on the empirical evaluation of StudyMe. Supplementary Figure S1. UML class diagram representing the data model of the Trial class and the classes it is composed of in StudyMe. The rectangles in the diagram represent classes and their properties. A line with a white triangle represents inheritance between classes, meaning the class below inherits the properties of the class above and that its objects are used in place of the upper class. A line with a black diamond at the end represents that the objects of the class on the end with a diamond are composed of objects of the class on the other end. Numbers on the lines, the multiplicities, represent how many objects are involved in the composition. One-to-one multiplicities are omitted from the diagram. Supplementary Figure S2. Overview of the steps of the iterative development process of StudyMe. [file 13063_2022_6893_MOESM1_ESM.pdf]

Supplementary Information

Supplementary Text S1: Details on the empirical evaluation of StudyMe

In the following, we provide more details on the results of the empirical evaluation of StudyMe from the user testing with 13 participants.

When analyzing the instructions that participants provided for their custom interventions in the created trials, it was apparent that each participant was able to specify how the intervention would be conducted, for example the person that defined an intervention with the name “cycling” wrote “cycle to university or on the way back home”. The participants used the settings of StudyMe to successfully define reminders for their interventions and measures at different times and frequencies. The four participants with sleep-related goals used daily reminders. Three of them (P2, P6, P13) set their intervention reminders to times in the evening between 6:00 p.m. and 10:30 p.m. The fourth person (P4) set the reminders for his intervention “eat dinner before 7pm” to noon. All four set their measure reminders to times in the morning between 6:30 a.m. and noon. Additionally, others set their reminders at different intervals, e.g., P12 who defined to go swimming every three days. 2 of the 13 participants changed the default schedule.

Qualitative feedback was gathered in addition to the SUS score results reported in the main manuscript. When asked what participants liked about the app, many participants mentioned aspects related to the flexibility and guidance that the app offers. They liked that they could create their own experiment. One participant stated that the app “allows me to experiment more than one way to reach my goal” and another that “it’s very flexible”. With respect to guidance, participants stated that they liked the app’s “structured approach”, “guided setup”, and “well explained process”. One participant wrote that it “feels empowering to get a support framework for self-testing”. Another liked the “recommendations for studies and actions” as well as the fact that the app uses statements with gaps that she can fill when creating a trial. Other positive remarks regarded “the reminder”, “good defaults”, and the app’s design which was described as “professional-looking” and “extremely clean”. It was also explicitly described as user-friendly, and “fast, easy and straight-forward to use”. With regard to what could be improved in StudyMe, some participants noted that there were “too many questions in the beginning”, that “setting notifications was cumbersome” and that it was “odd having to create separate reminders to collect data”. Seemingly contrary to this, one person said that she would actually like “more options to select dates”. While the app was designed to reduce the number of explanations needed to understand and create a trial, a few participants mentioned that they would want “more information on what a study is”, explanations on “implications of changing settings” or “how the results would be calculated”, and more clarity on the terms alternating and counterbalanced that are used under the advanced trial schedule settings in the app. Some suggestions were made that could inspire future versions of the app to include designing the app like a game, the ability “to do interventions together with a friend/family member” as well as recommendations about scheduling and trial settings depending on the selected intervention.

Supplementary Figures

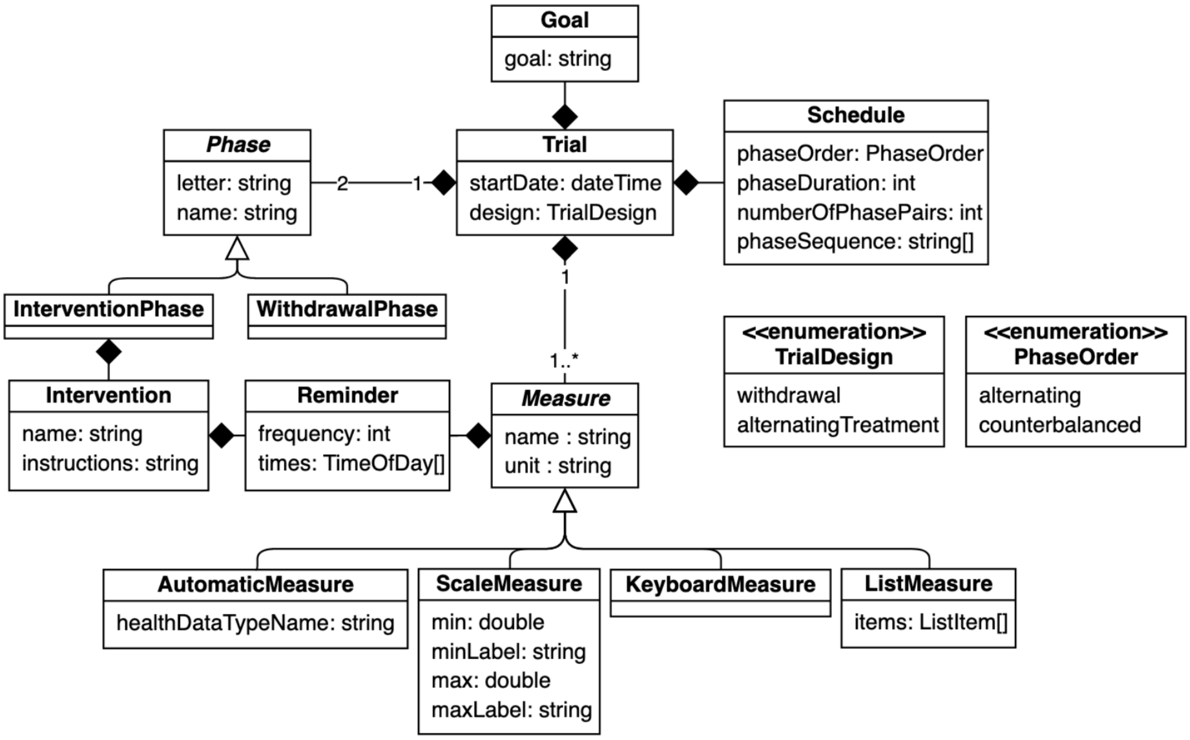

Supplementary Figure S1. UML class diagram representing the data model of the Trial class and the classes it is composed of in StudyMe.
The rectangles in the diagram represent classes and their properties. A line with a white triangle represents inheritance between classes, meaning the class below inherits the properties of the class above and that its objects are used in place of the upper class. A line with a black diamond at the end represents that the objects of the class on the end with a diamond are composed of objects of the class on the other end. Numbers on the lines, the multiplicities, represent how many objects are involved in the composition. One-to-one multiplicities are omitted from the diagram.

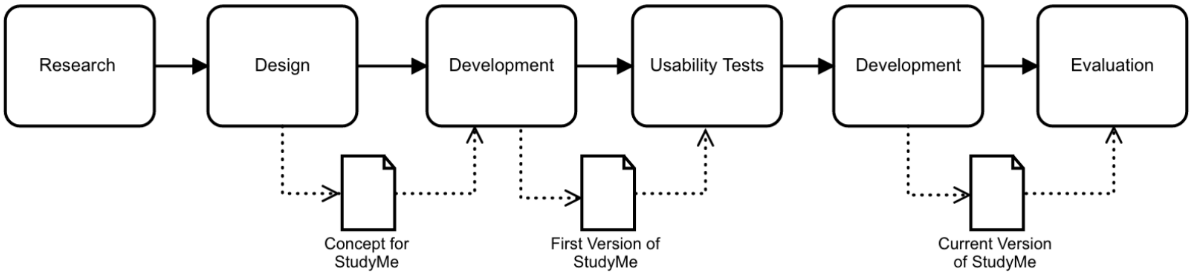

Supplementary Figure S2. Overview of the steps of the iterative development process of StudyMe.

Supplementary Video

Supplementary Video S1. Screen capture illustrating all steps in the StudyMe Health app in onboarding, generating, and running a trial.
